# Serotonin enhances neurogenesis biomarkers, hippocampal volumes, and cognitive functions in Alzheimer’s disease

**DOI:** 10.1186/s13041-024-01169-4

**Published:** 2024-12-18

**Authors:** Ali Azargoonjahromi, For the Alzheimer’s Disease Neuroimaging Initiative

**Affiliations:** Shiraz University of Medical Sciences, Shiraz, Iran

**Keywords:** Serotonin, Cognitive function, Alzheimer’s disease, Neurogenesis, Neuroplasticity, Gray matter volume, Brain structural volume

## Abstract

Research on serotonin reveals a lack of consensus regarding its role in brain volume, especially concerning biomarkers linked to neurogenesis and neuroplasticity, such as ciliary neurotrophic factor (CNTF), fibroblast growth factor 4 (FGF-4), bone morphogenetic protein 6 (BMP-6), and matrix metalloproteinase-1 (MMP-1) in Alzheimer’s disease (AD). This study aimed to investigate the influence of serotonin on brain structure and hippocampal volumes in relation to cognitive functions in AD, as well as its link with biomarkers like CNTF, FGF-4, BMP-6, and MMP-1. Data from 133 ADNI participants with AD included cognitive assessments (CDR-SB), serotonin measurements (Biocrates AbsoluteIDQ p180 kit, UPLC-MS/MS), and neurotrophic factors quantified via multiplex proteomics. Gray matter volume changes were analyzed using Voxel-Based Morphometry (VBM) with MRI. Statistical analyses employed Pearson correlation, bootstrap methods, and FDR-adjusted p-values (< 0.05 or < 0.01) via the Benjamini–Hochberg procedure, alongside nonparametric methods. The analysis found a positive correlation between serotonin levels and total brain (r = 0.229, p = 0.023) and hippocampal volumes (right: r = 0.186, p = 0.032; left: r = 0.210, p = 0.023), even after FDR adjustment. Higher serotonin levels were linked to better cognitive function (negative correlation with CDR-SB, r = −0.230, p = 0.024). Notably, serotonin levels were positively correlated with BMP-6 (r = 0.173, p = 0.047), CNTF (r = 0.216, p = 0.013), FGF-4 (r = 0.176, p = 0.043), and MMP-1 (r = 0.202, p = 0.019), suggesting a link between serotonin and neurogenesis and neuroplasticity. However, after adjusting for multiple comparisons and controlling for confounding factors such as age, gender, education, and APOE genotypes (APOE3 and APOE4), none of the correlations of biomarkers remained statistically significant. In conclusion, increased serotonin levels are associated with improved cognitive function and increased brain volume. However, associations with CNTF, FGF-4, BMP-6, and MMP-1 were not statistically significant after adjustments, highlighting the complexity of serotonin’s role in AD and the need for further research.

**Graphical Abstract:**

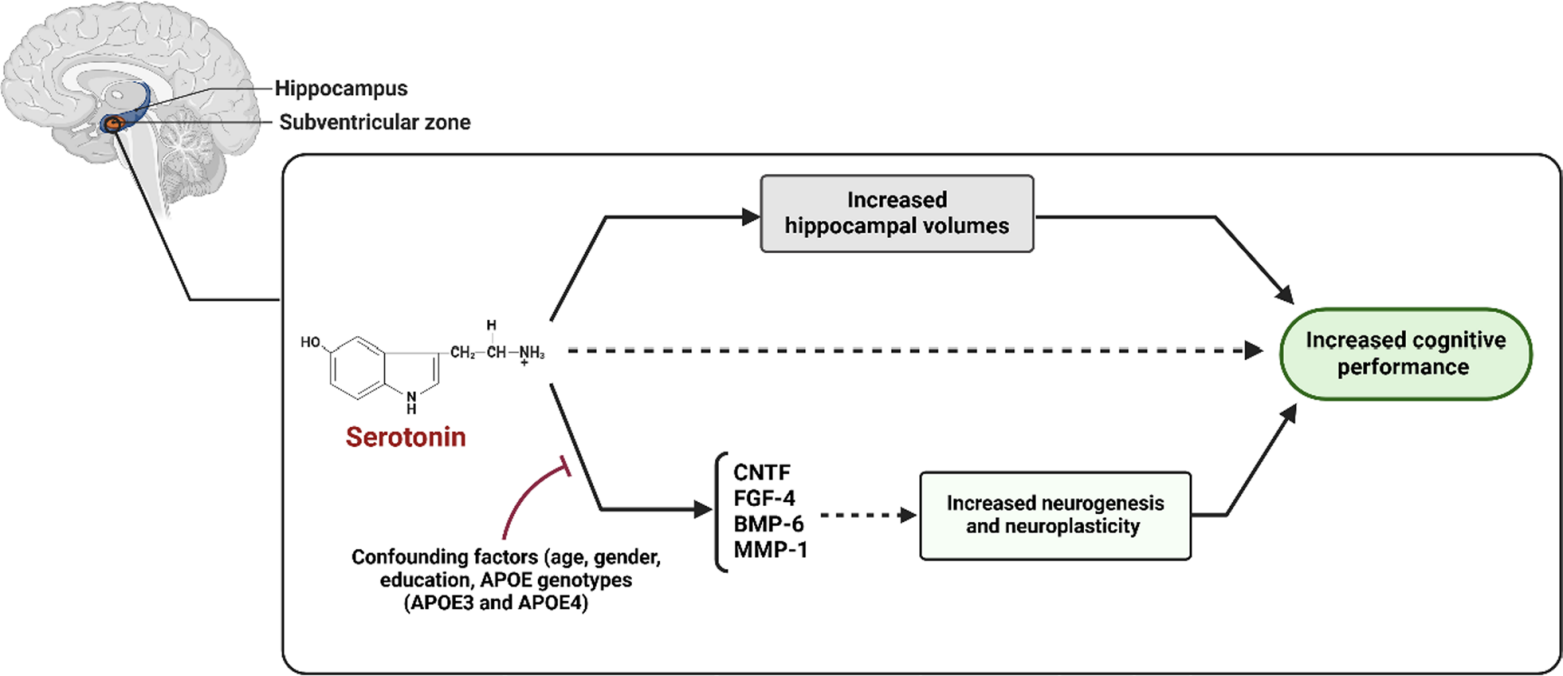

## Introduction

Serotonin (5-hydroxytryptamine, 5-HT) is a vital neurotransmitter that regulates mood, promotes neurogenesis, and supports neuroplasticity, particularly in the hippocampus [1–4], which is essential for learning and emotional regulation [5, 6]. Known as the “feel-good” chemical, serotonin is predominantly found in the brain and gastrointestinal tract, with about 90% located in the gut. It is synthesized from the amino acid tryptophan and influences various physiological functions, including mood regulation, bowel movement control, blood vessel constriction, appetite, sleep, and cognition [7, 8]. Notably, serotonin’s role in regulating neurogenesis and synaptic plasticity makes it a crucial focus in research on mood disorders like depression [9], as well as neurocognitive diseases such as Alzheimer’s disease (AD) [10].

Research has shown that serotonin induces neurogenesis, the formation of new neurons, primarily in the dentate gyrus (DG) of the hippocampus and the subventricular zone (SVZ) [11–14]. Indeed, elevated serotonin levels, often achieved through antidepressants such as selective serotonin reuptake inhibitors (SSRIs), have been demonstrated to enhance the generation of new neurons in these areas [15, 16]. Accordingly, serotonin and its receptors, including 5-HT1A, 5-HT1B, and 5-HT2, are crucial for promoting the growth of neural progenitor cells, which ultimately leads to the formation of new neurons [1, 12, 13, 17–19]. For instance, the activation of the 5-HT1A receptor has been shown to enhance neuron production in both the DG and SVZ; likewise, the 5-HT2A and 5-HT2C receptors influenced neurogenesis in various brain regions, underscoring serotonin’s diverse roles in neuronal development [11].

Despite such findings, a study found that lowering central serotonin levels in adulthood, achieved by depleting serotonergic neurons or inactivating serotonin synthesis, enhances adult hippocampal neurogenesis [20]. Similarly, a surprising finding in a transgenic rat model (TetO-shTPH2) suggested that reduced serotonin levels may actually promote increased neurogenesis, potentially serving as a compensatory mechanism in response to stress or injury. This challenges the prevailing view that higher serotonin levels consistently enhance neurogenesis [21]. Thus, such complexities highlight the nuanced role of serotonin in regulating neurogenesis under various conditions, including stress and neurodegeneration.

Beyond neurogenesis, serotonin can enhance neuroplasticity—the brain’s ability to form new neural connections—by interacting with brain-derived neurotrophic factor (BDNF), which supports neuron growth and synapse formation [14, 22, 23]. Notably, this interaction strengthens synaptic connections in the hippocampus, promotes neurogenesis and stress resilience—as shown in both human and animal studies—and plays a crucial role in cognitive functions due to increased BDNF expression [23–26]. Noteworthy, regarding serotonin’s role in other neurotrophic and nerve growth factors such as ciliary neurotrophic factor (CNTF), fibroblast growth factor 4 (FGF-4), bone morphogenetic protein 6 (BMP-6), and matrix metalloproteinase-1 (MMP-1), there has been, to our knowledge, no research conducted to clarify the specific interactions and regulatory mechanisms between serotonin and these factors concerning neurogenesis, neuroplasticity, and metabolic regulation. Therefore, further studies are essential to understand how serotonin influences these neurotrophic factors and their potential implications for neurodevelopment and neurodegenerative disorders. This current study aimed to address this gap.

In AD, the serotonin system was found to influence on key pathological processes such as β-amyloid and tau protein aggregation. Evidence suggested that targeting serotonin receptors may not only improve cognitive function but also address the root causes of AD-related dementia [10]. However, s evidence from zebrafish models revealed that serotonin can negatively regulate BDNF expression [27], which contrasts with other studies showing that serotonin and BDNF work synergistically to enhance neuroplasticity and neurogenesis [22–26]. This discrepancy suggests that serotonin may behave differently in neurodegenerative diseases like AD compared to typical neuroplastic or antidepressant mechanisms. This study aimed to explore the interaction between serotonin levels and neurotrophic factors and biomarkers related to neurogenesis and neuroplasticity, which has yet to be elucidated.

The former studies underscored the critical role of serotonin in regulating brain structure and function, particularly gray matter volume (GMV) and hippocampal volume. A positive correlation existed between serotonin receptor binding, especially the 5-HT1A receptor, and GMV, indicating these receptors influence brain integrity [28]. Likewise, in psychiatric conditions, altered serotonin receptor densities are linked to decreased GMV and hippocampal volumes, impaired neurogenesis, and structural brain changes, thereby contributing to mood disturbances and cognitive deficits [29, 30]. Of note, genetic variants, such as His452Tyr in the 5-HT2A receptor, were linked to reduced GMV in the hippocampus and poorer memory performance, highlighting the genetic influence on serotonin’s effects [31]. However, the correlation between 5-HT1A receptor binding and GMV was absent in individuals with autism spectrum disorder [32]. Overall, these findings emphasize serotonin’s multifaceted role in brain structure and function, warranting further investigations.

This study aimed to investigate, to the best of knowledge for the first time, how serotonin influences brain structure and hippocampal volumes, along with their connections to cognitive functions in AD. Besides, it sought to explore the relationship between serotonin and neurotrophic factors and biomarkers, such as CNTF, FGF-4, BMP-6, and MMP-1, pertained to neurogenesis and neuroplasticity.

## Methods and materials

Data for this article were obtained from the Alzheimer’s Disease Neuroimaging Initiative (ADNI) database (www.adni.loni.usc.edu), launched in 2003 by Principal Investigator Michael W. Weiner, MD. ADNI aims to determine if MRI, PET, biological markers, and clinical assessments can track the progression of MCI and early AD. The study recruited 133 participants who met the ADNI criteria for the AD group.

### Cognitive assessment

Among the three cognitive assessments—CDR, ADAS-Cog, and MMSE—this study specifically utilized the CDR-SB to establish correlations with other factors. In contrast, both the MMSE and ADAS-Cog were employed to provide a broader analysis of the participants’ overall cognitive functions. This distinction allows for a nuanced understanding of cognitive impairments in the context of AD, highlighting the unique role of each assessment tool in the evaluation process.

The clinical dementia rating (CDR) [33] scale was used to assess the overall severity of dementia symptoms across multiple domains, including memory, orientation, judgment, and personal care. The evaluation process involved a semi-structured interview with the participant and an interview with an informant, typically a family member or caregiver. Based on these interviews, scales ranged from 0 (no impairment) to 3 (severe impairment). The ratings from each domain were then combined to produce a global CDR score, which classifies participants into categories ranging from normal cognition, to mild cognitive impairment or dementia [34]. The CDR-sum of boxes (CDR-SB) score ranges from 0 to 18, with higher scores indicating greater cognitive and functional impairment. A score of 0 reflects no impairment, while scores between 0.5 and 18 represent varying levels of mild to severe dementia [35].

The Alzheimer’s disease assessment scale-cognitive subscale (ADAS-Cog) [36, 37] was specifically designed to measure the severity of cognitive symptoms associated with AD. It includes a series of cognitive tests that assess various domains such as memory, language, and praxis (motor coordination). Participants were asked to perform tasks like word recall, object naming, and copying geometric figures. The total score on the ADAS-Cog can range from 0 to 70, with higher scores indicating more severe cognitive impairment. This longitudinal tracking was crucial for understanding how cognitive decline progresses in individuals with MCI or AD [37].

The mini-mental state examination (MMSE) [38] is a widely used screening tool for assessing cognitive function, primarily in older adults. It comprises 30 questions that evaluate various cognitive domains, including orientation, attention, memory, language, and visuospatial skills. The MMSE score ranges from 0 to 30, with higher scores indicating better cognitive function. A score of 24 or lower typically suggests cognitive impairment, with lower scores often correlating with greater severity of dementia. The MMSE is commonly used in clinical settings to help diagnose and monitor cognitive decline [39, 40].

### Serotonin measurement

The measurement of serotonin levels in human serum samples was carried out using the Biocrates AbsoluteIDQ p180 kit, which quantifies over 180 metabolites, including serotonin. The process began with sample preparation, where 10 µL of serum was added to each well of a 96-well plate containing an internal standard for accurate quantitation. The plate was then dried under nitrogen, followed by derivatization with phenyl isothiocyanate to label biogenic amines, including serotonin. The samples were eluted with 5 mM ammonium acetate in methanol for extraction and diluted with 40% methanol in water for UPLC analysis.

Serotonin was analyzed using UPLC-MS/MS, with separation achieved through a Waters Acquity UPLC system using a C18 column. A gradient of 0.1% formic acid in water and acetonitrile was used, and detection occurred in Multiple Reaction Monitoring (MRM) mode with a Xevo TQ-S triple quadrupole mass spectrometer. The transition from precursor to product ions was monitored, with stable isotope-labeled standards ensuring accuracy. Data processing was done using TargetLynx^™^ software, with final analysis in Biocrates MetIDQ^™^ software. Calibration standards and quality control (QC) measures, including Golden West Serum, NIST SRM-1950 plasma, and a Study Pool QC, were incorporated to maintain accuracy. The QC pool was injected before and after every batch to check for potential batch effects, ensuring reliable results.

### Neurotrophic and growth factors measurement in plasma samples

In the ADNI, the measurement of neurotrophic and growth factors in plasma involved stringent QC procedures. QC measures included spiking blank human plasma with extracts from cell cultures expressing individual analytes, with low, medium, and high QC levels established for nearly all analytes and run in duplicate on 15 plates. A total of 1,065 500 μL EDTA plasma samples were processed, collected from participants after overnight fasting, within 120 min to ensure sample integrity.

The laboratory employed a multiplex targeted proteomics approach for quantifying analytes, which allowed for simultaneous measurement of multiple proteins in a single sample. Samples were randomized for analysis at the RBM facility, which remained blind to the clinical data. Both baseline and one-year follow-up samples were included, targeting individuals with MCI and AD. QC results were carefully monitored, with analytes showing CV values above 25% flagged for caution. The methodology addressed potential discrepancies such as outliers and samples below the least detectable dose (LDD). Statistical analyses adhered to a predefined plan, highlighting the importance of consulting trained statisticians for accurate interpretation.

### Neuroimaging technique (Voxel-Based Morphometry (VBM))

Voxel-based morphometry (VBM) using MRI data is a technique applied for the quantitative analysis of brain structural differences. VBM focuses on detecting regional volume changes in gray matter by analyzing the voxel-wise comparison of brain anatomy across different subjects, particularly in the context of neurodegenerative diseases like AD [41].

VBM involves several critical preprocessing steps to ensure accurate and reliable comparison of brain structures across subjects. First, high-resolution T1-weighted MRI images undergo denoising and bias correction to reduce noise and correct intensity variations. The brain images are then segmented into gray matter, white matter, and cerebrospinal fluid (CSF) compartments using a Bayesian framework. After segmentation, spatial normalization aligns the images to a standardized space (typically MNI), allowing for comparability across subjects. Finally, the segmented gray matter images are smoothed with a Gaussian kernel, improving statistical power and accommodating anatomical variability. VBM analysis is then performed using voxel-wise comparisons across different groups (e.g., AD patients versus controls), often using statistical parametric mapping (SPM) software. Statistical analysis follows, adjusting for multiple comparisons to ensure the reliability of results. This standardized pipeline ensures consistent, large-scale analysis of brain structure in neurodegenerative diseases.

### Statistical analysis

All statistical analyses were performed using Python (version 3.9.20) and IBM SPSS Statistics (version 27). Descriptive statistics, including means, standard deviations, and percentages, were calculated for the demographic characteristics and cognitive scores of the participants. The analysis utilized Pearson correlation and bootstrap methods to assess the relationships between variables, ensuring robust estimates of correlation coefficients with minimal bias and variability. To account for multiple comparisons and reduce the risk of false positives, p-values were adjusted using the Benjamini–Hochberg procedure for controlling the false discovery rate (FDR), with thresholds set at < 0.05 or < 0.01 for statistical significance. Additionally, nonparametric methods were employed to provide further validation of the results, enhancing the reliability of the findings in the presence of non-normal data distributions or small sample sizes.

## Results

A total of 133 participants diagnosed with AD were included in the study, comprising 79 males and 54 females. Participants’ ages ranged from 56 to 90 years, with a mean age of 76.20 years (SD = 7.39). Cognitive function was measured using multiple tools: the CDR-SB (mean = 4.88, SD = 4.14), the MMSE (mean = 22.11, SD = 4.28), and the ADAS-Cog (mean = 17.02, SD = 7.22). (Table 1).

**Table 1:** Demographic characteristics of the participants

Variable	N	Mean (SD)	Min	Max
Age (Y/O)	N/A	76.20 (7.39)	56	90
Handedness	N/A	1.08 (0.28)	1	3
- Right-handed	123	N/A	N/A	N/A
- Left-handed	10	N/A	N/A	N/A
Gender	N/A	1.41 (0.49)	1	2
- Males	79	N/A	N/A	N/A
- Females	54	N/A	N/A	N/A
Education	N/A	15.20 (3.04)	8	20
CDR-SB	N/A	4.88 (4.14)	0	18
MMSE	N/A	22.11 (4.28)	5	30
ADAS-Cog	N/A	17.02 (7.22)	3	43
APOE 4 carrier (positive/negative)	71/62	N/A	N/A	N/A
APOE 3 carrier (positive/negative)	87/46	N/A	N/A	N/A

### Serotonin level correlated with total brain volume and bilateral hippocampal volumes

The findings revealed correlations between serotonin levels and various brain volume measurements, including total brain volume and left and right hippocampal VBM. There was a significant positive correlation between serotonin and total brain volume (r = 0.179, p = 0.039), as well as between serotonin and right hippocampal VBM (r = 0.181, p = 0.037). A moderate positive correlation was observed between serotonin and left hippocampal VBM (r = 0.217, p = 0.012), also statistically significant. Besides, total brain volume showed positive correlations with both left hippocampal VBM (r = 0.172, p = 0.048) and right hippocampal VBM (r = 0.203, p = 0.019). Notably, left and right hippocampal VBM are strongly correlated (r = 0.870, p < 0.001). The results, supported by bootstrap confidence intervals and standard errors, suggested positive relationships between serotonin and brain volume metrics, particularly in the hippocampal regions. (Table 2).

**Table 2:** Correlations between serotonin level and various brain volume measurements

Variables	Serotonin	Total Brain Volume	Left Hippo VBM	Right Hippo VBM
Serotonin	1	0.179* (p = 0.039)	0.217* (p = 0.012)	0.181* (p = 0.037)
Total Brain Volume	0.179*	1	0.172* (p = 0.048)	0.203* (p = 0.019)
Left Hippocampal VBM	0.217*	0.172*	1	0.870** (p < 0.001)
Right Hippocampal VBM	0.181*	0.203*	0.870**	1

^*^Correlation is significant at the 0.05 level (2-tailed). **Correlation is significant at the 0.01 level (2-tailed). Values in bold are statistically significant

After adjustment for age, gender, education, and APOE genotypes (APOE3 and APOE4), the results showed significant positive correlations between serotonin levels and total brain volume (r = 0.229, p = 0.023), left hippocampal volume (r = 0.210, p = 0.023), and right hippocampal volume (r = 0.186, p = 0.032) (Table 3).

**Table 3:** Correlations between serotonin levels and brain volumes after adjustment for confounding factors

Variables	Correlation	FDR adjusted P-value
Total brain volume	0.229	0.023
Left hippocampal VBM	0.210	0.023
Right hippocampal VBM	0.186	0.032

### Serotonin level improved cognitive functions measured by CDR-SB

The findings showed the correlation between serotonin levels and CDR-SB scores in 133 participants. The Pearson correlation coefficient of -0.198 indicated a negative relationship, meaning higher serotonin levels are associated with lower CDR-SB scores, which reflect greater cognitive functions. This correlation was statistically significant (p = 0.023), suggesting a less than 5% probability that the result was due to chance. The sum of squares for serotonin was 21.593, and for CDR-SB, it was −25.287, indicating their interrelatedness.

Covariance values indicated the degree to which two variables change together. A covariance of 0.164 for serotonin meant that as serotonin levels increase, there was a tendency for the values of the other variable (CDR-SB) to change positively. Conversely, a covariance of −0.192 for CDR-SB suggested that as CDR-SB scores increase (indicating more cognitive impairment), serotonin levels tended to decrease.

Bootstrap analysis provided insights into the stability of these estimates. A bias of 0 for serotonin indicated that the estimate of its relationship with CDR-SB was unbiased, while a bias of −0.004 for CDR-SB suggested a slight negative adjustment. Standard errors reflected the variability of these estimates: a standard error of 0 for serotonin indicated no variability in the estimate, while a standard error of 0.067 for CDR-SB indicated some level of variability in the estimate for that variable. Overall, these results highlighted a positive significant relationship between serotonin levels and cognitive function, suggesting serotonin’s potential role in cognitive functions (Table 4). After adjustment for age, gender, education, and APOE genotypes (APOE3 and APOE4), the results also showed the same: A significant negative correlation between serotonin levels and cognitive impairment, as indicated by the CDR-SB (r = −0.230, FDR-adjusted p = 0.024).

**Table 4:** Correlations between serotonin level and cognitive functions measured by CDR-SB

Variable	Serotonin	CDR-SB
Pearson Correlation	1	−0.198*
Significance (2-tailed)		0.023
Sum of Squares and Cross-products	21.593	−25.287
Covariance	0.164	−0.192
Bootstrap Bias	0	−0.004
Standard Error	0	0.067

^*^Correlation is significant at the 0.05 level (2-tailed). Values in bold are statistically significant

### Total brain volume and bilateral hippocampal volumes correlated with cognitive functions measured by CDR-SB

The findings revealed correlations between the CDR-SB score, total brain volume, and VBM measures of the left and right hippocampus in participants. Higher CDR-SB scores, indicating greater cognitive impairment, were significantly correlated with reductions in total brain volume (r = −0.223, p = 0.010), left hippocampal volume (r = −0.246, p = 0.004), and right hippocampal volume (r = −0.308, p < 0.001), with a stronger relationship observed in the right hippocampus. Bootstrap analysis, used for verification, showed minimal bias and confirmed the significance of these correlations. The results showed that after FDR adjustment, the correlations between CDR-SB and brain volumes were weakened, but still significant; the correlations decreased in strength, with total brain volume (r = −0.162, p = 0.062), left hippocampal volume (r = −0.190, p = 0.043), and right hippocampal volume (r = −0.257, p = 0.009) (Table 5).

**Table 5:** Correlations between various brain volume measurements and cognitive functions measured by CDR-SB

Variables	CDR-SB (Before FDR-adjusted)	Bias	Std. Error	CDR-SB (After FDR-adjusted)
CDR-SB	1	0	0	1
Total brain volume	−**0.223** (p = 0.010)	0.001	0.080	−0.162 (P = 0.062)
Left hippocampus	−**0.246** (p = 0.004)	0.004	0.081	−**0.190 (P = 0.043)**
Right hippocampus	−**0.308** (p < 0.001)	0.003	0.085	−**0.257 (P = 0.009)**

Values in bold are statistically significant

### Serotonin level correlated with various neurotrophic and nerve growth factors involved in neurogenesis and neuroplasticity

**Fig. 1:**
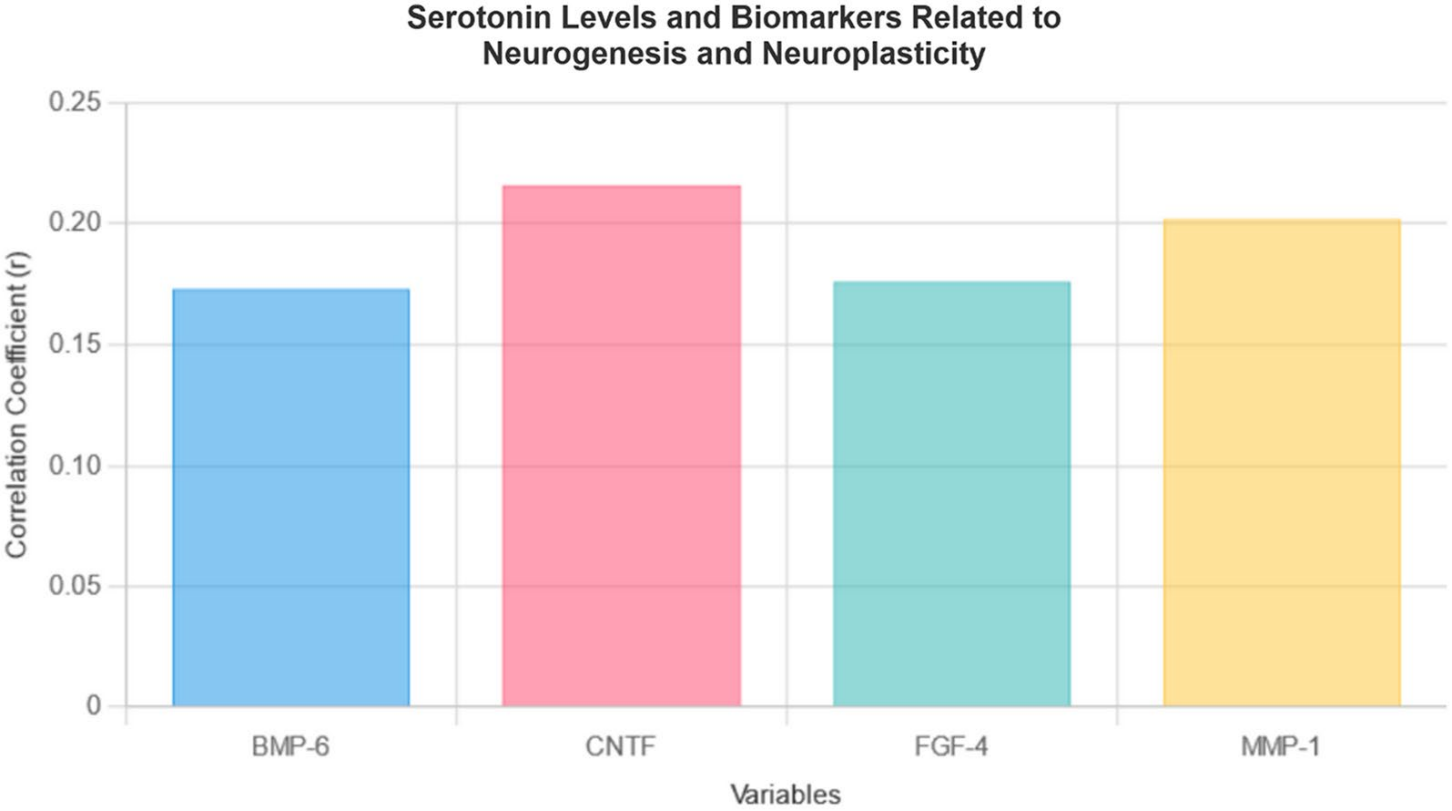
The bar chart presents the correlation coefficients of key biomarkers—BMP-6 (0.173), CNTF (0.216), FGF-4 (0.176), and MMP-1 (0.202)—associated with serotonin levels. These biomarkers play significant roles in neurogenesis and neuroplasticity, indicating their potential impact on brain health and development

The findings showed that serotonin has significant positive correlations with BMP-6 (r = 0.173, p = 0.047), CNTF (r = 0.216, p = 0.013), FGF-4 (r = 0.176, p = 0.043), and MMP-1 (r = 0.202, p = 0.019), suggesting that higher serotonin levels were associated with increased levels of these factors. BMP-6 strongly correlated with CNTF (r = 0.750, p < 0.001), FGF-4 (r = 0.734, p < 0.001), and MMP-1 (r = 0.446, p < 0.001), indicating significant co-regulation. Besides, CNTF and FGF-4 were strongly linked (r = 0.714, p < 0.001), and both showed positive correlations with MMP-1. However, after adjusting for multiple comparisons, the correlations between serotonin levels and these biomarkers (BMP-6, CNTF, FGF-4, and MMP-1) were no longer statistically significant. (Table 6) (Fig. 1).

**Table 6:** Correlations between serotonin level and various neurotrophic and nerve growth factors

Variables	Correlation	p-value	Correlation (After FDR-Adjusted)	FDR-adjusted p-value
BMP-6	0.173*	0.047	0.055	0.530
CNTF	0.216*	0.013	0.132	0.210
FGF-4	0.176*	0.043	0.085	0.444
MMP-1	0.202*	0.019	0.071	0.478

*BMP-6* Bone Morphogenetic Protein 6, *CNTF* Ciliary Neurotrophic Factor, *FGF-4* Fibroblast Growth Factor 4, *MMP-1* Matrix Metalloproteinase-1. Values in bold are statistically significant

^*^Correlation is significant at the 0.05 level (2-tailed)

## Discussion

The study on AD patients uncovered several significant relationships that emphasize the role of serotonin in brain health and cognitive function. A key finding, for the first time, was the positive correlation between serotonin levels and various biomarkers and neurotrophic factors, including CNTF, FGF-4, BMP-6, and MMP-1; however, after adjusting for multiple comparisons, these correlations were no longer statistically significant. Our confounders included age, gender, education level, and APOE genotypes (APOE3 and APOE4). These factors are involved in neurogenesis and neuroplasticity, suggesting that higher serotonin levels may stimulate their production, thereby promoting brain resilience and supporting cognitive health.

In addition, the research revealed a significant negative correlation between serotonin levels and cognitive impairment, as measured by the CDR-SB. Higher serotonin levels were associated with better cognitive function, suggesting a protective role against cognitive decline in AD and emphasizing its importance in cognitive health.

The study also found a positive association between serotonin levels and brain volume. This means that participants with higher serotonin levels exhibited increased total brain volume and larger hippocampal volumes, suggesting that elevated serotonin may help preserve or enhance brain structure. Moreover, the study demonstrated that reductions in brain volume, particularly in the hippocampus, were linked to greater cognitive impairment, further reinforcing the idea that maintaining brain and hippocampal size is crucial for cognitive preservation in AD patients.

Overall, the findings emphasized the interconnected roles of serotonin, brain structure, cognitive function, biomarkers, and neurotrophic factors in AD. They also suggested potential therapeutic strategies that focus on boosting serotonin levels to support brain health and enhance cognitive performance.

The findings of this study are consistent with earlier research emphasizing serotonin’s vital role in maintaining brain health and cognitive function. Both this research and prior studies demonstrated a clear link between higher serotonin levels and better cognitive performance, suggesting that serotonin plays a crucial role in enhancing cognitive outcomes. This connection highlights serotonin’s significance beyond its traditional function as a neurotransmitter, positioning it as a central component in the neurobiological mechanisms that support cognitive health [42–47].

Serotonin plays a crucial role in both physiological and cognitive functions by modulating neural activity through its widespread receptor subtypes. It influences key brain regions like the hippocampus and prefrontal cortex, which are essential for learning, memory, and decision-making [47–51]. The serotonergic system interacts with other neurotransmitters, such as dopamine, acetylcholine, and glutamate, contributing to cognitive processes [47]. In disorders like schizophrenia [52–56] and AD [10, 57–61], disrupted serotonergic pathways were linked to cognitive deficits, including impaired memory and reasoning. Thus, therapeutic strategies targeting serotonin receptors, such as 5-HTR3 and 5-HTR4, showed potential in improving cognition by regulating neurotransmitter release and reducing pathological markers like amyloid-β in AD [10, 43, 62, 63].

In addition to serotonin, other neurotransmitters like acetylcholine and dopamine can play a significant role in cognition and neuroprotection, particularly in the context of AD. Acetylcholine, through muscarinic receptors, is crucial for memory and attention, and its deficits contribute to cognitive decline in AD [64, 65]. Dopamine, acting on D1 and D2 receptors, supports motivation, attention, and executive function, with dysfunction in AD impairing working memory, attention, and goal-directed behavior [66]. These neurotransmitter systems do not operate in isolation; there is significant crosstalk between them. For example, dopamine and acetylcholine interact to modulate cognitive processes like attention and learning, with acetylcholine enhancing dopaminergic signaling in the prefrontal cortex. Disruption in this interplay can lead to the cognitive disturbances seen in AD, where the balance between excitation and inhibition in the brain is compromised. Together, these neurotransmitter systems are crucial for maintaining cognitive function, and their disruption in AD contributes to the disease’s hallmark cognitive impairments [67, 68].

The current findings expand previous knowledge by highlighting serotonin-linked biomarkers—CNTF, FGF-4, BMP-6, and MMP-1—that have been largely underexplored in earlier studies. These biomarkers are crucial for neurogenesis and synaptic plasticity [69–75], shedding light on potential mechanisms through which serotonin may influence cognitive health and brain resilience. However, the precise pathways by which serotonin increases these biomarkers remain unclear. While there is evidence that serotonin affects the expression of neurotrophic factors like BDNF [23, 76], its interactions with CNTF, FGF-4, BMP-6, and MMP-1 are not yet fully understood.

CNTF is a neurotrophic factor crucial for neuron survival and differentiation, primarily activating the JAK/STAT pathway [77–79] to maintain neuronal health and induce neurogenesis, particularly in conditions like AD [80–82]. FGF-4 similarly promotes the proliferation and differentiation of neural progenitor cells through FGF receptors and the MAPK pathway, supporting neurogenesis and cognitive resilience [71, 83–85]. BMP-6 may contribute to neuronal development and synaptic remodeling via Smad signaling, enhancing cognitive adaptability [86–89]. MMP-1 plays also a key role in extracellular matrix remodeling, enabling structural changes in synapses critical for synaptic plasticity [75, 90]. In short, serotonin’s influence on CNTF, FGF-4, BMP-6, and MMP-1 supports neurogenesis, neuronal survival, and synaptic plasticity, fostering cognitive resilience. However, the precise mechanisms by which serotonin mediates these effects remain unclear, requiring further research to fully understand its role in neurodegenerative diseases.

It is of note that some former findings highlighted the increased expression of BMP6 in the hippocampus of both human AD patients and APP transgenic mice, indicating its potential role in disrupting neurogenesis. For instance, qRT-PCR analysis revealed elevated BMP6 mRNA levels, which were corroborated by immunoblotting and immunohistochemical methods. The accumulation of BMP6 protein around amyloid plaques links its overexpression to amyloid-β pathology. In vitro studies further demonstrated that Aβ elevates BMP6 expression, subsequently inhibiting neural progenitor cell proliferation without inducing toxicity. These results suggest that Aβ-driven BMP6 upregulation may impair hippocampal neurogenesis, contributing to AD progression [91, 92]. Normalizing BMP6 levels could present a therapeutic strategy to mitigate neurogenic deficits in AD [92]. Similarly, another study found that increased BMP signaling in aged mice inhibits neural progenitor cell proliferation, but reducing BMP signaling restores neurogenesis, highlighting potential targets for treating age-related cognitive decline [93]. However, the significance of neurogenesis in cognitive function, particularly in the aging brain, remains unclear, as many AD-affected regions, like the neocortex, are non-neurogenic; thus, further investigation is needed to determine how such biomarkers can promote neurogenesis and potentially benefit AD patients.

The study found that higher serotonin levels in AD patients were positively associated with increased total brain and hippocampal volumes, indicating serotonin’s role in preserving brain structure. This aligns with earlier studies, though conducted on healthy individuals or other diseases, that emphasized serotonin’s influence on GMV and hippocampal size. Specifically, serotonin receptor binding, particularly 5-HT1A, was positively correlated with GMV, indicating its role in maintaining brain integrity [28]. In psychiatric conditions, altered serotonin receptor densities were linked to reduced GMV, impaired neurogenesis, and cognitive deficits [29, 30]. Genetic factors, like the His452Tyr variant in the 5-HT2A receptor, were also linked to reduced hippocampal volume and poorer memory performance [31]. However, no such correlation was found in individuals with autism spectrum disorder, highlighting variability in serotonin’s effects across conditions [32]. These findings underscore serotonin’s complex role in brain structure and cognitive function, calling for further research.

Of note, SSRIs are widely used to treat mood disorders such as depression and anxiety, but recent research highlights their role in promoting neurogenesis and synaptic plasticity, particularly in the hippocampus, a brain region critical for memory and emotional regulation. SSRIs function by inhibiting the serotonin transporter (SERT), thereby increasing serotonin availability in the synaptic cleft [94, 95]. This elevated serotonin activates several intracellular signaling pathways, including the MAPK/ERK pathway, which promote neuronal growth and survival [96, 97]. A key mediator in this process is BDNF, a protein that fosters neurogenesis by stimulating the proliferation of neural progenitor cells and their differentiation into mature neurons [14, 98, 99].

In addition, SSRIs have been shown to enhance long-term potentiation (LTP), a key process of synaptic strengthening where repeated neuronal activation improves the efficiency of synaptic transmission [100]. This effect is partly mediated by increased glutamatergic activity, which supports synaptic strengthening, and modulation of GABAergic function, which helps maintain the balance between excitation and inhibition in neural circuits. Over time, chronic SSRI treatment induces structural brain changes, such as increased hippocampal volume, linked to enhanced neurogenesis and improved cognitive function [101–103]. These neuroplasticity-promoting effects likely underlie the therapeutic benefits of SSRIs in mood disorders and hold promise for treating conditions with impaired neuroplasticity, such as neurodegenerative diseases.

The study had several strengths that enhanced our understanding of serotonin’s role in AD. One key strength was its focus on specific brain regions, particularly the hippocampus, which is crucial in AD pathology. This targeted approach added significant relevance to the findings. Besides, the study explored the connections between serotonin levels and neurotrophic factors like BMP-6, CNTF, and FGF-4. This study added a valuable layer of molecular insight, suggesting that serotonin might have influenced neurogenesis and neuroplasticity in the context of neurodegeneration. The use of bootstrap analysis also increased the reliability of the results, providing more robust estimates of the relationships among the variables studied.

However, there were notable limitations in this research that needed to be considered. The cross-sectional design made it difficult to establish causal relationships between serotonin levels and brain structure or cognitive function, limiting the ability to determine the direction of these associations. Further, the study had a relatively small sample size, which raised concerns about the statistical power of the findings. The sample’s demographic composition did not fully represent the broader AD population, particularly regarding gender balance and age distribution, which could have limited the applicability of the results. Moreover, the presence of non-normal distributions in variables like CDR-SB, ADAS-Cog, and MMSE could have impacted the accuracy of parametric statistical tests, potentially leading to biased conclusions. Lastly, the study did not include genetic analyses, leaving out the possibility of genetic factors, apart from APOE genotypes, influencing serotonin’s effects on brain structure and function. Future studies should address these limitations by adopting longitudinal designs, increasing sample size, ensuring demographic diversity, applying appropriate statistical methods, and incorporating genetic analyses to achieve more convergent findings.

## Conclusion

The findings revealed that serotonin plays a crucial role in influencing brain structure and cognitive functions in individuals with AD. Specifically, higher serotonin levels were associated with larger brain and hippocampal volumes, which in turn correlated with better cognitive performance; even after adjustments for some confounding factors like age, gender, education, and APOE genotypes (APOE3 and APOE4). Furthermore, although the initial positive associations between serotonin and biomarkers related to neurogenesis and neuroplasticity, such as CNTF, FGF-4, BMP-6, and MMP-1, were observed, these correlations were no longer statistically significant after adjustments for multiple comparisons. This suggests that the role of serotonin in enhancing neurogenesis and neuroplasticity, particularly in AD, may require further investigation with larger, more controlled studies to confirm its therapeutic potential. In short, these insights underscore the importance of serotonin in the context of neurodegeneration and highlight areas for further research into its therapeutic potential.

## Data Availability

The data used in this research was obtained from Alzheimer’s disease Neuroimaging Initiative (ADNI) and is available with permission to all researchers.

## Abbreviations

AD: Alzheimer’s disease
DG: Dentate gyrus
SVZ: Subventricular zone
BDNF: Brain-derived neurotrophic factor
CNTF: Ciliary neurotrophic factor
FGF-4: Fibroblast growth factor 4
BMP-6: Bone morphogenetic protein 6
MMP-1: Matrix metalloproteinase-1
GMV: Gray matter volume
CDR: Clinical dementia rating
ADAS-Cog: Alzheimer’s disease assessment scale-cognitive subscale
MMSE: Mini-mental state examination
QC: Quality control
VBM: Voxel-based morphometry
CSF: Cerebrospinal fluid
